# Pre-operative localization of solitary pulmonary nodules with computed tomography-guided hook wire: report of 181 patients

**DOI:** 10.1186/s13019-016-0404-4

**Published:** 2016-01-16

**Authors:** Matthieu Hanauer, Jean Yannis Perentes, Thorsten Krueger, Hans-Beat Ris, Pierre Bize, Sabine Schmidt, Michel Gonzalez

**Affiliations:** Division of Thoracic Surgery, Centre Hospitalier Universitaire Vaudois, 1011 Lausanne, Switzerland; Division of Radiology, Centre Hospitalier Universitaire Vaudois, Lausanne, Switzerland

## Abstract

**Background:**

Video-assisted thoracic surgery (VATS) is currently performed to diagnose and treat solitary pulmonary nodules (SPN). However, the intra-operative identification of deep nodules can be challenging with VATS as the lung is difficult to palpate. The aim of the study was to report the utility and the results of pre-operative computed tomography (CT)-guided hook wire localization of SPN.

**Methods:**

All records of the patients undergoing CT-guided hook wire localization prior to VATS resection for SPN between 2002 and 2013 were reviewed. The efficacy in localizing the nodule, hook wire complications, necessity to convert VATS to thoracotomy and the histology of SPN are reported.

**Results:**

One hundred eighty-one patients (90 females, mean age 63 y, range 28–82 y) underwent 187 pulmonary resections after CT-guided hook wire localization. The mean SPN diameter was 10.3 mm (range: 4–29 mm). The mean distance of the lesion from the pleural surface was 11.6 mm (range: 0–45 mm). The mean time interval from hook wire insertion to VATS resection was 224 min (range 54–622 min). Hook wire complications included pneumothorax requiring chest tube drainage in 4 patients (2.1 %) and mild parenchymal haemorrhage in 11 (5.9 %) patients. Migration of the hook wire occured in 7 patients (3.7 %) although it did not affect the success of VATS resection (nodule location guided by the lung puncture site). Three patients underwent additional wedge resection by VATS during the same procedure because no lesion was identified in the surgical specimen. Conversion thoracotomy was required in 13 patients (7 %) for centrally localized lesions (6 patients) and pleural adhesions (7 patients). The mean operative time was 60 min (range 18–135 min). Pathological examination revealed a malignant lesion in 107 patients (59 %). The diagnostic yield was 98.3 %.

**Conclusion:**

VATS resection for SPN after CT-guided hook wire localization for SPN is safe and allows for proper diagnosis with a low thoracotomy conversion rate.

## Background

The solitary pulmonary nodule (SPN) is defined by a single, well circumscribed radiographic opacity, that measures less than 3 cm and is completely surrounded by aerated lung parenchyma. The incidental findings of SPN are currently increasing because of a more generalized use of chest computed tomography (CT) in screening trials of high risk patients or during follow up in oncologic patients [1]. Almost 50 % of these nodules are malignant. Therefore, a rapid and precise histological diagnosis is necessary [2]. The effectiveness of conventional procedures (trans-bronchial biopsy or CT-guided fine needle biopsy) may be occasionally limited by the localization or by the small size of the nodule [3]. When the diagnosis cannot be obtained with these less invasive techniques, excisional biopsy by video-assisted thoracic surgery (VATS) is generally proposed for definitive diagnosis and therapeutic procedure. VATS is associated with low morbidity and short hospitalization [3, 4]. Nevertheless, intra-operative identification of the lesion may be problematic due to the difficulty to palpate the lung during thoracoscopy. This issue holds especially true for deeply located small lesions, ground glass opacities and lesions localized in emphysematous lung or surrounded by dense pleural adhesions [5].

Various techniques for SPN localization have been described, including finger palpation [5], intra-operative ultrasound [6–10], CT-guided insertion of localizer (hook wire [11–17], microcoils [18], methylene blue [19], Lipiodol [20], or radionuclides [21]) with success rates as high as 100 % [22]. The hook wire technique showed a varied success rate ranging from 58 to 97.6 % in various series with relatively higher failure rate due to wire dislodgement reaching up to 47 % [4]. Minor complications such as asymptomatic pneumothorax or parenchymal hemorrhages are commonly associated, while serious events are unfrequently reported [17]. In 2002, we successfully introduced the use of CT-guided hook wire program to localize SPN. We initially reported our preliminary results in 45 patients [14]. The current study follows-up on our updated experience of 12 years and focuses on the utility of pre-operative CT-guided hook wire localization in our center.

## Methods

One hundred eighty-one consecutive patients undergoing VATS resection for SPN with pre-operative CT-guided hook wire localization between January 2002 and December 2013 were retrospectively reviewed.

Before referral for excisional biopsy, all patients were evaluated in an interdisciplinary setting. Pre-operative CT-guided hook wire localization was indicated when thoracic surgeons considered that detection of SPN during VATS would be difficult. In case of solid nodules, hook wire localization was indicated for small subpleural (less than 10 mm) or deeply located nodules situated at a distance of >10 mm from the visceral pleura. Pre-operative localization was also required for each subpleural cavitary lesion or ground glass opacity. All patients signed a written informed consent and each case was discussed between the thoracic surgeon and radiologist prior to VATS.

The device consists of a circular hook wire connected to a 50-cm-long suture thread (Ariadne’s Thread, Laurane Medical®, Le Pradet, France). The hook is contained within a 20-gauge, 10-cm-long needle and regains its circular configuration of 8 mm diameter after deployment providing reliable anchoring into the nodule.

**Fig. 1:**
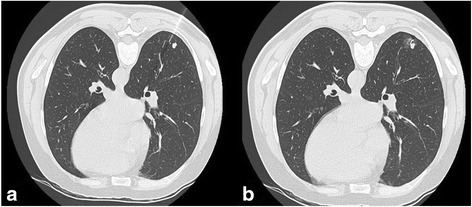
A 66-year-old patient with a solitary pulmonary nodule of unknown origin and situated in the right lower lobe, thus scheduled for VATS. (**a**) After CT-acquisition in prone position the 20-gauge needle is introduced (**b**), followed by the deployment of the hook wire, accidently causing a focal, small and asymptomatic pneumothorax

The procedure took place at the day of the surgery, immediately before surgery. First, a non-enhanced CT-scan acquisition was performed (120 kV, 180 mA, pitch 1.375, table speed 55 mm/0.5 s, reconstructed axial slice of 2.5 mm/2 mm) to confirm the exact localization of the SPN. The patient’s position on the CT table depended on the shortest distance between the skin and the SPN and simultaneously permitted a safe access with the hook wire. After determination of the puncture site, local anesthesia was performed and, under CT-guidance, the hook wire system was gradually inserted into the lung parenchyma, followed by the final deployment, further controlled by CT-scan to confirm the exact location of the hook wire and absence of immediate complications as pneumothorax or parenchymal bleeding (Fig. 1).

In case of technical difficulty or very small size of the SPN, the hook wire was deployed as close as possible to the nodule. After device removal, the thread remained outside of the patient was covered with sterile gauze and left loose enough in order to follow lung collapse during VATS procedure. The patient was then directly transferred to the operating room.

VATS surgery was performed under general anesthesia. Intubation consisted in a double-lumen endobronchial tube using an immediate single lung ventilation to avoid positive pressure in the punctured lung, and thus, potential risk of tension pneumothorax. Surgical resection was undertaken using a standardized three port approach with the camera placed in the seventh intercostal space on the anterior axillary line. Then, the hook wire was visualized going through the chest wall and anchoring into the nodule. At that time, we try to confirm the presence of the nodule by finger palpation. Additional incisions were performed for wedge resection by endoscopic staplers’ devices, in the seventh intercostal space on the posterior axillary line and in the fourth intercostal space on the anterior axillary line. All surgical specimens were extracted in a protective bag to prevent chest wall implantation of malignant disease. If frozen section examination revealed lung cancer, lobectomy with mediastinal lymph node dissection was carried out by thoracotomy (before September 2010) or by VATS (after September 2010). In these cases, conversion thoracotomy was not considered as failure of localization. Otherwise, the operation was completed, thoracic trocars were removed and chest tubes inserted.

The files of the patients were analyzed in view of patients’ characteristics, efficacy of pre-operative localization and complications of the, hook wire, operative time, necessity of conversion thoracotomy, SPN histology and post-operative complications.

## Statistics

The descriptive statistics for continuous variables were presented as mean ± SD. Multivariate analysis was performed by logistic regression to determine the risk of malignancy based on pre-operative clinical and radiological findings. *P*-values less than 0.05 were considered as significant. All statistical analyses were performed with the STATA software (version 11.1; Texas USA).

## Results

Between January 2002 and September 2013, 181 patients (91 males/90 females, mean age 63 years, range 28–82 years) underwent 187 resections of SPN localized pre-operatively by CT-guided hook wire. Six patients underwent 2 hook wire insertions simultaneously because of 2 nodules in the same lung. The mean diameter of the SPN at CT was 10.3 mm (range 4–29 mm). One hundred twenty-three (67.9 %) nodules were less than 10 mm in maximal diameter. The mean distance from the SPN to the pleura was 11.6 mm (range 0–45 mm). The CT-feature of SPN showed solid nodules in 168 patients (93 %), pure ground-glass opacities in 7 (4 %) and cavitary lesions in 3 (3 %). Pre-operative PET-CT was performed in 112 patients. Nodules presented high uptake on PET-CT in 83 of them (74 %).

The mean time interval from hook wire insertion to VATS resection was 224 min (range 54–622 min). The hook wire was inserted in the right upper lobe in 48 patients (25.5 %), middle lobe in 18 (9.6 %), right lower lobe in 36 (19.3 %), left upper lobe in 49 (26.2 %) and left lower lobe in 35 (18.7 %). During procedure, patients were positioned on the CT-scan table in supine position (*n* = 87, 48 %), prone position (*n* = 83, 46 %) and lateral position (*n* = 11, 6 %). All these data are summarized in Table 1.

**Table 1:** Patients’ characteristics, and radiological features and localization of the solitary pulmonary nodules

Characteristic	Value
Patients	181
Procedure	187
Sex ratio (m/f)	91/90
Mean age (y)	63 (range 28–82)
Mean nodule size (mm)	10.3 (range 4–29)
Nodule size <10 mm	123 (67,9 %)
Mean distance from lesion to pleural surface (mm)	11,6 (range 0–45)
Mean time interval from hook wire insertion to VATS resection (min)	224 (range 54–622)
Aspect of the lesion	
Solid	169 (93 %)
Ground glass opacity	7 (4 %)
Cavitary	6 (3 %)
Localization of the hook wire	
RUL	48 (25,5 %)
RML	18 (9,6 %)
RLL	36 (19,3 %)
LUL	49 (26,2 %)
LLL	35 (18,7 %)
Position of the patient during hook wire placement	
Supine	87 (48 %)
Prone	83 (46 %)
Lateral	11 (6 %)

**Table 2:** Complications related to hook wire placement

Type of complication	Number of patients
Pneumothorax	71 (38 %)
Asymptomatic	67 (35,9 %)
Symptomatic	4 (2,1 %)
Parenchymal bleeding	11 (5,9 %)
Hemothorax	0
Dislodgement	7 (3,7 %)
Absence of lesion in the surgical specimen	11 (6 %)

Dislodgment of the hook wire occurred in 7 patients (3.7 %) (Table 2). However, VATS resection was successfully accomplished in all these patients by locating the lung puncture site. Three patients (1.7 %) had no identifyable lesion in the surgical specimen despite closed localization of the hook wire. Nevertheless, all these 3 patients underwent successful additional wedge resection by VATS during the same procedure (taking the first staple line in toto). Unplanned conversion thoracotomy occurred in 13 patients (7 %): 6 because of centrally located lesions not amenable to wedge resection by VATS (3 of them close to the pulmonary artery), and 7 with thick pleural adhesions requiring thoracotomy for adhesiolysis. Thirty-four patients underwent completion lobectomy during the same procedure by either thoracotomy (*n* = 14) or thoracoscopy (*n* = 20). Mean operative time for VATS resection was 60 min (range 18–135 min).

**Fig. 2:**
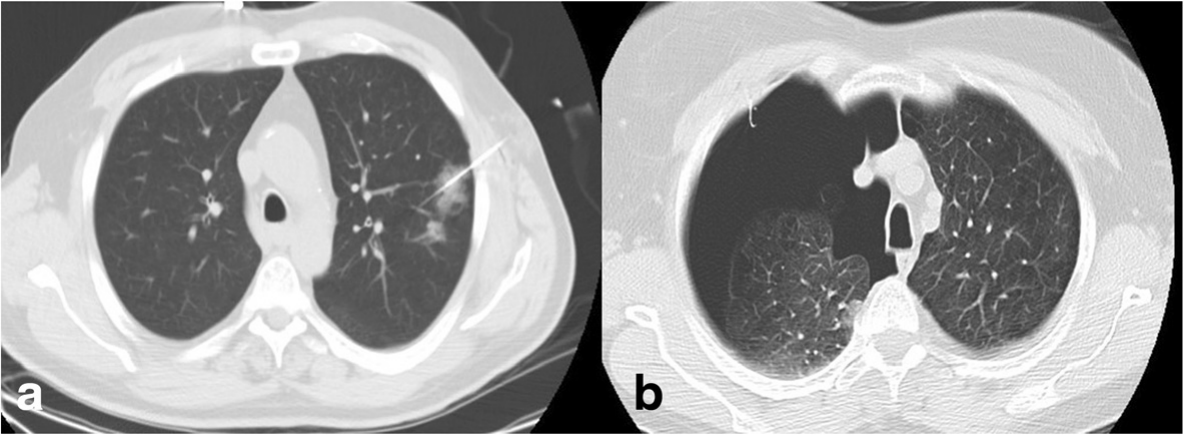
**a** Axial CT-image shows parenchymal bleeding of the left upper lobe located around the needle of the hook wire after insertion. **b** Axial CT-image demonstrates an important pneumothorax of the right lung occurring after hook wire insertion requiring chest tube drainage

After hook wire placement, CT-scan revealed the presence of pneumothorax in 71 patients of which 67 (35.9 %) were small in size with no respiratory symptoms while 4 (2 %) required prompt small bore chest tube insertion by radiologist (Fig. 2). Interestingly, all four patients presented important emphysematous bullae and poor pulmonary functions. CT-scan showed parenchymal bleeding in 11 patients (5.9 %) without no clinical consequence (Fig. 2). No hemothorax or pulmonary air embolism were reported. The mean hospitalization length of stay was 4 days for wedge resection (range 1–50 days). *For patients undergoing lobectomy, the mean hospitalization was 8 days.”*

Table 3 summarizes results of the histological findings of the resected SPN. Malignant disease was found in 107 patients (59 %), among which primary NSCLC in 47 patients (25.6 %). The diagnostic yield was 98.3 % (3/184).

**Table 3:** Histological findings of the pulmonary nodules

Histological findings	Number of patients (%)
Malignant lesion	107 (59)
Adenocarcinoma	33
Squamous cell carcinoma	11
Large cell carcinoma	3
Carcinoid tumor	2
Lymphoma	3
Metastases	55
Benign lesion	74 (41)
Hamartoma	11
Granuloma	15
Inflammatory disease	18
Lung fibrosis	8
Lymph node	18
Adenomatous atypic hyperplasia	1

On a multivariate analysis in this series, we identified five pre-operative clinical and radiological factors related to malignancy (Table 4): age older than 60 years (OR: 2.40), previous history of malignancy (OR. 6.43), size of the nodule of more than 10 mm (OR: 3.61), nodule localized in the upper lobe (OR 3.61) and high uptake of the nodule on PET-CT imaging (OR: 5.81).

**Table 4:** Clinical predictor of malignancy for patient undergoing surgical biopsy of solitary nodules

Clinical feature	Number of patients	Number of malignancy (%)	OR (95 % CI)	*P* value
Male	91	51/91 (56 %)	0.70 (0.42–3.63)	0.39
>60 year-old	109	77/109 (70.6 %)	2.40 (1.59–3.63)	0.0001
Tobacco abuse	111	64/111 (57.7 %)	0.85 (0.46–1.57)	0.61
Prior malignancy	107	82/107 (76.6 %)	6.43 (3.33–12.4)	0.00001
Size > 10 mm	60	47/60 (78 %)	3.61 (1.95–6.61)	0.0001
Superior lobe	91	63/91 (69.2 %)	2.35 (1.28–4.31)	0.0052
PET-positive	82/110	66/82 (80.5 %)	5.83 (2.96–11.48)	0.00001

## Discussion

Solitary pulmonary nodules are a frequent incidental finding due, in part, to the increased number of CT-examinations performed for lung cancer or during the follow-up screening or past malignancy [1]. When pulmonary nodules are encountered especially with previous history of malignancy or tobacco use, histological analysis is mandatory to determine further treatment [2]. Generally, in cases of SPN >1 cm in size, diagnosis is initially attempted with less invasive techniques, such as CT-guided percutaneous needle biopsy or transbronchial biopsy [2]. However, even with refinement of the technique for bronchoscopy with ultrasound or electronavigation, both procedures allow definite diagnosis in less than 80 % of cases, especially in cases of SPNs that are less than 1 cm and located in non-favorable positions [2]. In the last decade, VATS has progressively gained acceptance for the management of SPN to provide a definite diagnosis, reassurance in case of benign lesions and sufficient material for molecular analysis, thus avoiding the drawbacks of thoracotomy. Moreover, VATS is now indicated for definite treatment in case of early stage NSCLC proceeding with lobectomy and lymphadenectomy when indicated [23]. The real benefit of VATS for the post-operative outcome is counterbalanced by the difficulty to locate SPN during the procedure. Simple palpation by finger introduced in the utility incision is generally sufficient for lesion of >2 cm located in the pleural surface [12]. Finger localization may be also difficult depending on the composition of the lesion (ground-glass opacity, or cavitary lesion), of the lung parenchyma (fibrosis, emphysema, strong adhesion) or the proximity of the bronchi [16]. Pre-operative localization is now routinely recommended for small lesion of less than 10 mm, located at a distance of more than 5 mm from the pleural surface due to associated high rate of conversion thoracotomy [5].

Several techniques have been developed to facilitate intra-operative localization of SPN during VATS [22]. Methylene blue injection carries the risk of spreading the colorant on the pleural surface or difficulty to identify the lesion in patients with extensive anthracotic pigments [19]. This technique has been associated with a rate of failure of 13 %. Specialized equipment, which is not currently available, such as CT-fluoroscopy [18, 20, 24] or gamma probe [21], is required in case of injection of specific radiotracer. Intra-operative ultrasound detection requires a specific flexible ultrasound and is operator dependent [6–10]. Localization of SPN is also limited by the presence of air which can be difficult to obtain in non-collapsed lung or in emphysematous patients. Formless abnormalities such as ground glass opacities or inflammatory lesions may be particularly difficult to visualize and may prolong the duration of the operation.

We previously published our preliminary experience with the use of CT-guided hook wires to localize SPN [14]. We reported that this system allowed precise and quick pre-operative localization of SPN with a low rate of complications. During VATS resection, complete excision of small and deeply localized lesions by endo-staplers may be improved by traction on the suture thread, facilitating SPN accessibility. This approach may also be an useful for localization of peripheral nodules when VATS resection is performed by less experienced surgeon*.* Seven patients (3.6 %) presented dislodgement of the hook wire without consequence due to the easy localization of the puncture site on pleural surface. Our rate of dislodgement is acceptable and consistent with the 5–8 % reported in the literature [11, 12, 16, 17]. Based on our experience, this complication may be reduced by maintaining an adequate length of wire cable outside the thorax, thus allowing the wire to follow the collapsed lung during selective ventilation or pneumothorax. We recommend also to place the hook at least 10 mm under the pleural surface to ensure good anchorage, due to increased risk of dislodgement in case of insertion distance from the pleura less than 5 mm [25].

Generally, hook wire insertion does not take much time.between 15 to 30 min per lesion [16, 17]. Furthermore, the vicinity of radiology unit and the possibility to perform the procedure just before the operation has made this technique convenient for the patient with a short delay until surgery. The mean delay between the hook wire placement and the operation was 224 min. This relatively long interval is explained by the fact that VATS resection is generally planned in the operating room in second or third position for organization issues of our operative theater. However, some centers proceed to insertion the day before surgery for convenience, without increased complication [17].

Conversion thoracotomy was necessary in our study in 13 patients. Six of them required thoracotomy to perform pulmonary resection due to central lesions. All of them presented a deeply localized lesion located at > 2.5 cm from the pleural surface. Retrospectively, VATS was probably not indicated in these patients. Seven patients required conversion due to severe pleural adhesions. Finally, no patient required conversion due to inadequate hookwire insertion or dislodgement.

Pneumothorax occurred after hook wire insertion in 71/181 patients (38 %). These pneumothoraces were minimal and not relevant, thus asymptomatic in 67 patients, but 4 out of them required chest tube insertion at the radiology unit. All of them had important emphysema or the nodule was located next to a emphysematous bulla, but all sustained VATS resection. In these cases, caution should be taken and hook wire should be inserted only if chest tube can be promptly inserted. The rate of pneumothorax reported previously in association with hook-wire insertions oscillates between 18 to 35 % with a majority of them considered as minor [11–13]. This rate is probably underestimated, because 68 % patients who had chest X-ray 30 min after hook wire presented a pneumothorax,as reported by Ichinose et al. [17].

Parenchymal bleeding is occasionally reported after hook wire insertion (13/181). Our series, was not complicated with any episode of hemoptysis neither related difficulty for surgical resection after parenchymal bleeding. The clinical impact of parenchymal bleeding after hook wire is thus not relevant.

Venous air embolism is a life threatening complication occasionally reported after hook wire insertion. However, this complication was not observed in our series.

The histological analysis of SPN confirms the high percentage of malignant disease and endorses the previously reported results in this respect.

Patient’s age and gender, history of smoking, especially current smokers, prior malignancy, radiological findings as size, morphology and location, and PET-CT hypercaptation are commonly admitted as malignancy criteria. We found that age older than 60 years, previous history of malignancy, size of the nodule of more than 10 mm, nodule localized in the upper lobe and high uptake of the nodule on PET-CT imaging were clinical predictor of malignancy.

## Conclusion

In conclusion, as SPN is a common pathology more often related to malignancy than of benign origin, thus it needs to be characterized early For this purpose, VATS resection after CT-guided hook wire localization for SPN is safe and allows for proper diagnosis with a low conversion thoracotomy rate.
